# TAAR1 in Addiction: Looking Beyond the Tip of the Iceberg

**DOI:** 10.3389/fphar.2018.00279

**Published:** 2018-03-27

**Authors:** Jian-Feng Liu, Jun-Xu Li

**Affiliations:** ^1^Department of Pharmacology and Toxicology, University at Buffalo, Buffalo, NY, United States; ^2^School of Pharmacy, Yantai University, Yantai, China

**Keywords:** TAAR1, drug addiction, dopamine, psychostimulants, relapse

## Abstract

Trace-amine associated receptor 1 (TAAR1) is the best-characterized member of the family of TAARs. TAAR1 is broadly expressed in the brain, especially within the monoaminergic systems. Evidence from electrophysiological and neurochemical studies evaluating the effects of genetic and pharmacological interventions on TAAR1 revealed that TAAR1 modulates transmission of monoamines, especially dopamine. TAAR1 agonists dampened drugs of abuse-induced dopamine accumulation. In general, TAAR1 agonists specifically inhibited the rewarding and reinforcing effects of drugs of abuse and drug-abuse related behaviors. Details of the mechanism of TAAR1 remain elusive; however, it is thought to be regulated by its interactions with D2 receptors. In addition, the alternative cellular mechanism such as an interaction between TAAR1 and D3 may also participate in the action of TAAR1 agonists. Further studies are required to investigate the role of TAAR1 in other drugs of abuse-related behaviors and the underlying neural mechanisms. Collectively, TAAR1 negatively modulates dopaminergic systems and dopamine-related behaviors and TAAR1 agonists are promising pharmacotherapy to treat drug addiction and relapse.

## Introduction

**Figure 1:**
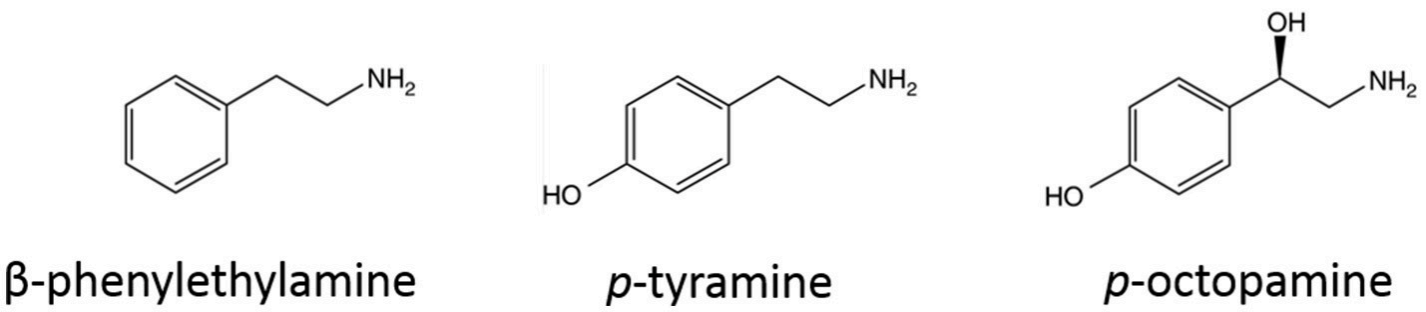
Structures of representative trace amines (endogenous agonists of trace amine-associated receptor 1).

The family of trace amine-associated receptors (TAARs) has 9 members (TAAR1-9) (Grandy, 2007). Among the TAARs, TAAR1 is the best characterized and studied one. The endogenous ligands of TAAR1 in mammalian tissue, trace amines, are a group of relatively low-expressed amines structurally similar to classic amines (structure of representative trace amines are listed in Figure 1; Grandy, 2007; Miller, 2011). For decades, trace amines were thought to be false neurotransmitters (Grandy, 2007). Trace amines have been thought to play important roles in psychiatric disorders, such as depression, schizophrenia, and attention deficit hyperactivity disorder (8115671; 12550748), but the absence of a targetable receptor for trace amines was a long hindrance to the field (Miller, 2011). In 2001, two studies from independent groups identified the family of trace amine-associated receptors (Borowsky et al., 2001; Bunzow et al., 2001). Since then, the literature about the function of TAARs, especially TAAR1, has been quickly accumulating.

TAAR1 is broadly expressed in the brain, which was identified by both genetic and biochemical strategies (Grandy, 2007). In the central nervous system, mRNA and protein of TAAR1 were identified to be expressed in the dopaminergic projections (ventral tegmental area, VTA, substantia nigra, SN, dorsal and ventral striatum), glutamatergic projections (frontal cortex, amygdala, subiculum), and serotonergic projections (dorsal raphe) (Miller, 2011; Grandy et al., 2016). Although the neuroanatomical expression of TAAR1 in the brain is well-known, the subcellular distribution of TAAR1 is still paradoxical due to the low expression of TAAR1 and lack of good quality antibody. By using virus-mediated overexpression strategy, it is demonstrated that the primary expression of TAAR1 is located intracellular (Xie and Miller, 2009), however, it is possible that unidentified membrane components mediate the transferring of TAAR1 to plasmid membrane. In the CNS, TAAR1 is suggested to exist in both pre- and post-synaptic neuronal components, as well as astrocytes (Xie and Miller, 2009; Cisneros and Ghorpade, 2014). The circuitry and subcellular expression pattern of TAAR1 in the CNS make it a reasonable candidate that possibly participates in mental disorders. To date, TAAR1 has been demonstrated to be involved in a broad range of neuropsychiatric disorders including schizophrenia, depression, bipolar disorder, Parkinson's disease, sleep disorders, drug abuse and addiction (Grandy et al., 2016).

Among the studied disorders in which TAAR1 plays a crucial role, drug addiction (especially stimulants addiction) is the best studied (Jing and Li, 2015; Pei et al., 2016). In recent years, a growing literature demonstrated that TAAR1 negatively modulates amphetamine-like stimulants- and cocaine-related behaviors (Table 1; Jing and Li, 2015). In this review, we will focus on the role of TAAR1 in regulating drug addiction and related underlying neural mechanisms.

**Table 1:** Modulation of TAAR1 activity on abuse-related behaviors of drugs.

**TAAR1 modulation**		**Treatment**	**Species**	**Behavior**	**Effect**	**References**
Full agonists	RO5166017	Cocaine	Mice	Cocaine-induced hyperlocomotion	↓	Revel et al., 2011, 2013b
			Rats	Expression of CPP	↓	Liu et al., 2016
				Cue- and priming-induced reinstatement of cocaine-seeking	↓	Liu et al., 2017
		DAT^−/−^	Mice	Spontaneous hyperlocomotion	↓	Revel et al., 2013b
	RO5256390	Cocaine	Mice	Cocaine-induced hyperlocomotion	↓	Revel et al., 2013b
			Rats	Cocaine-seeking after abstinence for 2 weeks	↓	Pei et al., 2014
				Cocaine self-administration	↓	Pei et al., 2015
				Cocaine-induced lowering of ICSS reward thresholds	↓	Pei et al., 2015
		Palatable food	Rats	Eating for a highly palatable sugary diet	↓	Ferragud et al., 2017
Partial agonists	RO5263397	Cocaine	Mice	Cocaine-induced hyperlocomotion	↓	Revel et al., 2013b
			Rats	Cocaine-induced hyperlocomotion	↔	Thorn et al., 2014a,b
				Expression of cocaine-induced sensitization	↓	
				Expression of cocaine-CPP	↓	Thorn et al., 2014b
				Development of cocaine-CPP	↔	
				Cue- and priming-induced reinstatement of cocaine-seeking	↓	
				Cocaine-induced lowering of ICSS reward thresholds	↓	Pei et al., 2015
		METH	Rats	Expression of METH-induced sensitization	↓	Jing et al., 2014
				Cue- and priming-induced reinstatement of METH-seeking	↓	
				METH self-administration	↓	
				Effects of METH in 5-CSRTT	↓	Xue et al., 2018
				Effects of METH in delay discounting	↔	
		Sucrose	Rats	Cue-induced reinstatement of sucrose-seeking	↔	Jing et al., 2014
	RO5073012	AMPH	Mice	AMPH-induced hyperlocomotion	↔	Revel et al., 2012
	RO5203648	Cocaine	Mice	Cocaine-induced hyperlocomotion	↓	Revel et al., 2012a; Pei et al., 2015
			Rats	Cocaine-induced hyperlocomotion	↓	
				Self-administration	↓	Pei et al., 2014
				Cocaine-seeking after abstinence for 2 weeks	↓	Cotter et al., 2015
				Priming-induced reinstatement of cocaine-seeking	↓	
		METH	Rats	Development of sensitization	↓	Cotter et al., 2015
				Cross-sensitized with METH	↑	
				Early phase of METH-induced hyperlocomotion	↓	
				Late phase of METH-induced hyperlocomotion	↑	
				Self-administration	↓	
		DAT^−/−^	Mice	Spontaneous hyperlocomotion	↓	Revel et al., 2012a
		Sucrose	Rats	Sucrose self-administration	↓	Cotter et al., 2015
TAAR1 knockout		AMPH	Mice	AMPH-induced hyperlocomotion	↑	Lindemann et al., 2008; Achat-Mendes et al., 2012
				Context-dependent sensitization	↑	Sukhanov et al., 2016
				Acquisition of CPP	↔	
				Reinstatement of CPP	↑	
		METH	Mice	METH-induced hyperlocomotion	↑	Achat-Mendes et al., 2012
				CPP	↑	
				METH-induced CTA	↓	Harkness et al., 2015
		Ethanol	Mice	Preference for ethanol in two-bottle choice	↑	Lynch et al., 2013
				Locomotion after ethanol administration	↓	
		Morphine	Mice	CPP	↔	Achat-Mendes et al., 2012
		Apomorphine	Mice	Climbing behavior and certain types of stereotypic behavior	↓	Sukhanov et al., 2014
				Apomophine-induced hypolocomotion	↔	
		Sucrose	Mice	Preference for sucrose in two-bottle choice	↔	Lynch et al., 2013
TAAR1 overexpression		AMPH	Mice	Hyperactivity	↓	Revel et al., 2012

*TAAR1, trace amine-associated receptor 1; CPP, conditioned place preference; CTA, conditioned taste aversion; 5-CSRTT, 5-choice serial reaction time task; AMPH, amphetamine; METH, methamphetamine; DAT, dopamine transporter. ↑, upregulation; ↓ downregulation; ↔ no change*.

## TAAR1 and psychostimulants-related behaviors

### TAAR1 and amphetamines

In 2001, Bunzow, et al. cloned functional rat TAAR1 (rTAAR1) in HEK293 cells (Bunzow et al., 2001). Functional assays showed that trace amines preferentially stimulated the TAAR1 to produce the second messenger cAMP. Considering the structural similarity of amphetamine to trace amines, they tested whether amphetamine analogs could stimulate TAAR1 (Bunzow et al., 2001). The results were intriguing, which showed that amphetamine, methamphetamine (METH), MDMA, and the major amphetamine metabolite para-hydroxyamphetamine (POHA) potentially stimulated cAMP production in the cells transferred with TAAR1 but not with an empty vector or with the human DA receptor (Bunzow et al., 2001). A later study confirmed the finding but also indicated that TAAR1 in mouse, rat, and human showed species-dependent stereoselectivity for amphetamines (Reese et al., 2007). These pharmacological profiles revealed that amphetamines are ligands of TAAR1.

Consistent with these, TAAR1 knockout (TAAR1-KO) mice showed hypersensitivity to amphetamine-induced hyperactivity, rearing behavior, extracellular dopamine (DA) and norepinephrine (NE) release in dorsal striatum (Wolinsky et al., 2007; Lindemann et al., 2008). TAAR1-KO mice revealed the increase in context-dependent conditioning to amphetamine (Sukhanov et al., 2016) and showed hypersensitivity to METH-induced hyperlocomotion and augmented METH-induced conditioned place preference (CPP) (Achat-Mendes et al., 2012). In 2012, Revel et al. generated an animal line (*Taar1* Tg mice) that overexpressed TAAR1 exclusively in the brain (Revel et al., 2012). To more clearly demonstrate the expression pattern of TAAR1 in this mouse line, we renamed the *Taar1* Tg mice as TAAR1 overexpression (TAAR1-OE) mice in this review. It would be expected that overexpression of TAAR1 in the brain would mimic the effects of TAAR1 activation by its agonists. Surprisingly, the spontaneous firing rate of dopaminergic neurons in TAAR1-OE mice was enhanced, which seems to be opposite to the finding that TAAR1 agonists reduced the firing rate of dopaminergic neurons (Revel et al., 2012). Accordingly, the basal levels of DA and NE in the nucleus accumbens (NAc) were augmented in TAAR1-OE mice. A behavioral analysis revealed that TAAR1-OE mice were less responsive to amphetamine-induced hyperactivity without affecting the general motor functions and behaviors (Revel et al., 2012). It should be noted that, in the TAAR1-OE mice, overexpression of TAAR1 is not limited to the cell population normally expressing *taar1* in WT animals. The electrophysiological and molecular changes in TAAR1-OE mice could be presumably mediated by the removal of GABAergic inhibition on the dopaminergic neurons (Revel et al., 2012), which may not be easy to interpret the TAAR1's function in WT animals. In addition, Harkness et al, showed that DBA/2J mice which carry a non-functional allele of *taar1* gene consumed more METH than C57BL/6J mice that express normal TAAR1 (Harkness et al., 2015). Taken together, these results from engineered mice lines revealed a fundamental role of TAAR1 in regulating amphetamines-induced addiction-related behaviors.

Several highly selective TAAR 1 ligands were recently developed. The selective TAAR1 partial agonist RO5263397 was the first TAAR1 agonist that was studied in amphetamines-related behaviors (Jing et al., 2014). Without affecting the spontaneous locomotion, RO5263397 significantly reduced the expression of METH-induced behavioral sensitization (Jing et al., 2014). It was also shown that another TAAR1 partial agonist RO5203648 prevented METH-induced behavioral sensitization (Cotter et al., 2015). Both RO5263397 and RO5203648 significantly attenuated METH self-administration. Moreover, in a rat model of drug relapse, RO5263397 decreased discrete cue- and priming-induced reinstatement of METH-seeking behavior (Jing et al., 2014). In addition, several evidence indicated that the inhibitory effects of TAAR1 activation were highly selective on rewarding and reinforcing effects of drugs. First, RO5203648 did not affect the self-administration of a natural reinforcer saccharin. Second, RO5263397 had no effect on the reinstatement of sucrose-seeking behavior. Third, RO5203648 alone did not maintain self-administration behavior using a substitution procedure in rats self-administering METH, indicating that RO5203648 itself had no reinforcing effect (Cotter et al., 2015). Taken together, these studies confirmed that TAAR1 is a promising pharmacological target for the intervention of addiction to amphetamines.

### TAAR1 and cocaine

Another psychostimulant that is reportedly regulated by TAAR1 is cocaine (Jing and Li, 2015). Cocaine is a non-selective competitive inhibitor of monoamine transporters and apparently is not a ligand of TAAR1 (Jonkman and Kenny, 2013). Based on the results that TAAR1 modulates the monoamine system in the brain (see details below), it is reasonable to examine the pharmacological effects of TAAR1 agonists on cocaine-related behaviors. The selective TAAR1 full agonist RO5166017 significantly decreased cocaine-induced hyperlocomotion in WT mice but not in TAAR1-KO mice (Revel et al., 2011). TAAR1 partial agonist RO5203648 dose-dependently reduced cocaine-induced hyperlocomotion and cocaine self-administration (Revel et al., 2012a). Consistent with these results, the TAAR1 full agonist RO5256390 and the partial agonist RO5263397 dose-dependently attenuated cocaine-induced hyperlocomotion in mice (Revel et al., 2013b). In a more systematic examination of cocaine-related behaviors, Thorn et al. (2014a,b) demonstrated that RO5263397 attenuated the induction and expression of cocaine-induced sensitization, expression of cocaine-induced CPP, and cue- and priming -induced reinstatement of cocaine-seeking behavior. Using a behavioral economic analysis, RO5263397 increased the elasticity of the cocaine demand curve, suggesting that RO5263397 could accelerate the decrease in cocaine consumption when the price increases. Pei et al. also showed that the partial TAAR1 agonist RO5203648 and the full agonist RO5256390 decreased cocaine self-administration and cocaine-seeking behaviors (Pei et al., 2014, 2015). In a procedure to evaluate cocaine's facilitation on the brain reward function, both RO5203648 and RO5256390 dose-dependently reverted cocaine-induced decrease in the thresholds of the intracranial self-stimulation (Pei et al., 2015).

To further understand how TAAR1 is involved in cocaine addiction, our recent study investigated the role of TAAR1 in cocaine-related reward memory by using a widely used drug reward memory paradigm, cocaine-induced CPP (Liu et al., 2016). Drug reward memory hijacks the same neural circuitry and cellular signaling pathways involved in normal and neutral reward memory system (Kauer and Malenka, 2007). Drug reward memory also includes the same memory process as other memories, such as acquisition/conditioning, consolidation, storage, expression/retrieval, and reconsolidation (Lu et al., 2006). The TAAR1 full agonist RO5166017 suppressed the expression while hindered the formation of the long-term memory of cocaine reward memory. RO5166017 had no effect on the reconsolidation of cocaine reward memory (Liu et al., 2016). Furthermore, repeated administration of RO5166017 before the extinction of cocaine self-administration had no long-lasting effect in the reinstatement of cocaine-seeking tests. Our previous study showed that the TAAR1 partial agonist RO5263397 had no effect on the acquisition of cocaine reward memory (Thorn et al., 2014a). These results strongly indicate that TAAR1 activation specifically inhibits the expression of cocaine reward memory.

## TAAR1 and other addiction-related behaviors

### TAAR1 and ethanol

In a standard two-bottle choice paradigm, TAAR1-KO mice consumed more ethanol than WT mice (Lynch et al., 2013). In contrast, TAAR1-KO and WT mice showed no difference in drinking from the sucrose bottle vs. water bottle, suggesting that TAAR1 is specifically involved in alcohol addiction but not naturally rewarding behavior (Lynch et al., 2013). Additionally, TAAR1-KO mice showed enhanced sedative-like effects induced by acute ethanol treatment in a loss of righting reflex paradigm and greater reduction in locomotor activity after ethanol treatment than WT mice (Lynch et al., 2013). These results, albeit preliminary, do suggest that TAAR1 is also involved in the rewarding and reinforcing effects of ethanol.

### TAAR1 and morphine

To the best of our knowledge, only one study examined the possible role of TAAR1 in modulating morphine-related behaviors. Achat-Mendes et al. compared the difference between WT and TAAR1-KO mice in morphine-induced CPP (Achat-Mendes et al., 2012). They showed that both TAAR1-KO and WT mice developed the same preference to morphine-paired side (Achat-Mendes et al., 2012). Moreover, no difference was found between TAAR1-KO and WT mice in the extinction or the reinstatement in morphine-induced CPP. Taken together, it seems that endogenous TAAR1 was not involved in morphine-induced CPP (Achat-Mendes et al., 2012). However, it should be noted that although TAAR1-KO mice did not show significant difference with WT mice in cocaine-induced hyperlocomotion, TAAR1 agonists significantly prevented cocaine-induced hyperlocomotion in WT animals (Revel et al., 2013b). Thus, it is possible that the modulation of TAAR1 activity could affect morphine-induced CPP in WT animals. Furthermore, these results from morphine-induced CPP could not rule out the role of TAAR1 in other morphine-related behaviors. For example, the deletion of the δ opioid receptor gene in mice preserved the reinforcing effects of morphine but disrupted morphine-induced CPP (Le Merrer et al., 2011). Also, the adenosine A_2A_ receptor plays different roles in opiate reinforcement and opiate-seeking behavior (Brown et al., 2009). More thorough studies are needed to delineate the potential role of TAAR1 in mediating addiction-related effects of opioids.

### TAAR1 and other drugs of abuse

So far, existing genetic and pharmacologic studies have suggested essential roles of TAAR1 in mediating the addiction-related effects of several drugs of abuse including amphetamines, cocaine, ethanol, and morphine. There are clear evidence indicating that, in general, TAAR1 activation negatively modulates the rewarding and reinforcing effects of most of the studied drugs of abuse (Jing and Li, 2015). Preclinical studies discussed above also suggest that selective TAAR1 agonists could be promising agents to treat drug addiction.

However, although the emerging studies from the past several years builds a general impression that we already gained plenty of knowledge about the pharmacology of TAAR1 and its role in the regulation of drugs of abuse, we may have only seen the tip of the iceberg.

The categorization of drugs of abuse typically includes stimulants, opiates, depressants, and hallucinogens (Akerele and Olupona, 2017), and the current understanding of the relationship between TAAR 1 and the different classes of abused drugs vary substantially. The following represents a simplified analysis. (1) Stimulants: Although the effects of TAAR1 agonists on amphetamines- and cocaine-related behaviors were well-studied, whether the addiction-related effects of other psychostimulants, particularly the major addictive component in tobacco (nicotine), could be regulated by TAAR1 are unknown (Bruijnzeel, 2017). (2) Opiates: The current knowledge about the involvement of TAAR1 in opioid addiction is extremely limited (only one study Lynch et al., 2013), which calls for more systematic studies. Opioids such as morphine and oxycodone are powerful painkillers but are highly addictive. Given the current knowledge regarding the inhibitory effects of TAAR1 agonists on psychostimulants addiction, it is crucial to systemically determine the role of TAAR1 in opioid-related behaviors. If TAAR1 agonists negatively regulates the addiction-related but not analgesic effects of opioids, then TAAR1 agonists could be useful adjuvants as a combination therapy with opioids to promote opioid-induced analgesia while working against the current opioid epidemic. (3) Depressants: There was only one study using TAAR1-KO mice suggesting that TAAR1 is also involved in certain pharmacological effects of ethanol (Lynch et al., 2013), and the effects of TAAR1 agonists on alcohol-related behaviors are unknown. Further studies are needed to clarify whether TAAR1 is involved in the regulation of the pharmacological effects of other depressants such as diazepam and other benzodiazepines (Tan et al., 2010). (4) Hallucinogens: Lysergic acid diethylamide (LSD) has been reported as a TAAR1 agonist (Bunzow et al., 2001). Treatment with a TAAR1 antagonist EPPTB significantly blocked the inhibitory effect of LSD on dopaminergic neurons (De Gregorio et al., 2016). To date, no functional study has been reported on the role of TAAR1 in any other hallucinogens. (5) Others: TAAR1 agonists reduced phencyclidine (PCP)-induced hyperlocomotion (Revel et al., 2013b), but other effects are unknown. In addition, whether TAAR1 could modulate effects of cannabis/marijuana are unknown. All the above is an essential void in the knowledge to truly understand the functional interaction between TAAR1 and drug addiction.

### TAAR1 and behavioral addiction

The concept of addiction does not only refer to drug addiction but also to non-drug related behavioral addiction (Alavi et al., 2012). The concept of behavioral addiction was first proposed by Peele and has been progressively refined over time (Peele, 1979). Although currently only one type of behavioral addiction, gambling addiction, is listed in the category of “Substance-related and Addictive Disorders” in DSM-V, other excessive/compulsive behaviors are also considered behavioral addiction such as sex and love addiction, internet addiction, shopping addiction, video game addiction, and food addiction (Grant and Chamberlain, 2016). The potential involvement of TAAR1 agonists in behavioral addiction is poorly understood.

In a recent study, Ferragud et al. examined the effects of a TAAR1 agonist on food addiction (Ferragud et al., 2017). They found that the TAAR1 full agonist RO5256390 prevented binge eating of highly palatable food in rats (Ferragud et al., 2017). RO5256390 also blocked the conditioned rewarding properties of palatable food and palatable food-seeking behavior (Ferragud et al., 2017). This study suggests the possibility that TAAR1 could also be involved in the regulation of behavioral addiction in the way that is similar to drug addiction. Such a possibility should not surprise anyone given that decades of research strongly support the notion that all addictive behaviors have largely overlapping neuroanatomical underpinnings, i.e., mesolimbic and mesocortical dopaminergic neurocircuitry. In this regard, more detailed studies are required to unveil the role of TAAR1 in other types of behavioral addiction.

### TAAR1 and negative reinforcement

The development of addiction are not only associated with the rewarding and positive reinforcing effects of the drugs of abuse, many of those drugs, particularly opioids and nicotine, also recruit the brain stress system and decrease the function of the brain reward system (Koob, 2017). These “dark-side” effects of drugs of abuse are commonly defined as negative reinforcing factors. Discontinued drug use could induce various negative responses such as chronic irritability, physical pain, emotional pain (i.e., hyperkatifeia), malaise, dysphoria, alexithymia, and loss of motivation for natural rewards (Koob, 2015). Negative reinforcement could be a critical factor that leads to repeated relapse after abstinence from drugs of abuse (Koob, 2015). The role of TAAR1 in the negative reinforcing effects of drugs is unknown, and the delineation of this role is an important piece of the puzzle.

## Neural mechanisms of TAAR1

### Neuroanatomical sites of action of TAAR1 agonists

As mentioned above, the expression of TAAR1 in the CNS shows an overlap with the rewarding circuitry in the brain, especially the dopaminergic system (Grandy, 2007). The major brain areas of the mesocorticolimbic dopaminergic system include the VTA, SN, dorsal striatum, ventral striatum/NAc, and prefrontal cortex (Melichar et al., 2001). Studies using genetic and systemic pharmacological interventions cannot address which of these brain areas are important for TAAR1's function.

Our recent study using intracranial microinjection technique investigated this question (Liu et al., 2017). The TAAR1 full agonist RO5166017 which has been shown to significantly block cocaine-induced hyperlocomotion and cocaine-induced CPP in previous studies was used in our study (Liu et al., 2017). RO5166107 was microinjected into several brain areas before cue- and priming-induced reinstatement of cocaine-seeking behavior in rats. The results showed that TAAR1 in theVTA, NAc, and prelimbic area of the medial prefrontal cortex (mPFC) are important for the reinstatement of cocaine-seeking behavior, whileTAAR1 in the SN, infralimbic area of the mPFC, and amygdala do not seem to be important for TAAR1 in mediating the reinstatement of cocaine-seeking behavior (Liu et al., 2017). These results suggest that TAAR1 within specific subregions of the mesocorticolimbic dopaminergic system plays a critical role in drug addiction.

Ferragud et al. also showed that microinjection of the TAAR1 full agonist RO5256390 into the infralimbic area of mPFC reduced food addiction (Ferragud et al., 2017). Importantly, exposure to high palatable food significantly reduced TAAR1 expression in the mPFC, which indicates that TAAR1 could be one of the biological substrates mediating the pathogenesis of food addiction (Ferragud et al., 2017). In addition, the discrepancy that TAAR1 in the different subregions of mPFC participates in cocaine and food addiction suggests that the neuroanatomical sites of TAAR1 in different types of addiction could be different.

### TAAR1 modulation of neurotransmitters and cellular cascades

The first study on the interaction between TAAR1 and dopamine transporter (DAT) showed that the function of TAAR1 was modulated by DAT (Miller et al., 2005). DAT is a critical molecule in the transmission of the neurotransmitter dopamine. In the presynaptic membrane, by pumping dopamine from the synaptic cleft back into the cytosol, DAT provides the primary neural mechanism of dopamine clearance from the synaptic cleft (Espana and Jones, 2013). It was found that coexpression of human DAT with rhesus monkey TAAR1 in HEK-293 cells significantly enhanced the activation of TAAR1 by amphetamine and MDMA, indicating that TAAR1 activation may be dependent on DAT. *In vitro* experiments further showed that TAAR1 modulates dopamine reuptake, which depends on the PKA and PKC activities (Miller et al., 2005). Furthermore, TAAR1 activation promotes the efflux of dopamine which was blocked by inhibition of DAT and PKC (Miller et al., 2005). *In vivo*, TAAR1 was shown to be coexpressed with DAT in a subset of dopaminergic neurons in the SN of rhesus monkeys and mice (Wolinsky et al., 2007).

Existing data suggest that the effects of TAAR1 on DAT's function could be mediated indirectly through the interaction between TAAR1 and D2 autoreceptors (Xie and Miller, 2007). TAAR1 activation by dopamine was blocked when TAAR1 and D2 receptors were co-expressed in cells, indicating that TAAR1 activation can be regulated by D2 receptors (Xie and Miller, 2007). It was also shown that the common biogenic amines (DA, NE, and 5-HT) can modulate the function of monoamine transporters by TAAR1 and monoamine autoreceptors in transfected cells and mouse brain synaptosomes (Xie et al., 2008). These results suggest that TAAR1 is a modulator of DAT, which then regulates dopamine reuptake (Xie and Miller, 2007, 2009). By using fast-scan cyclic voltammetry (FSCV) experiments, it was demonstrated that the evoked DA release was higher in the NAc of TAAR1-KO mice as compared to their WT counterparts (Leo et al., 2014). The notion that TAAR1 interacts with D2 autoreceptors was also supported in *in vivo* settings. *In vivo* microdialysis study showed that the extracellular DA was increased in the NAc of TAAR1-KO mice as compared to the WT mice (Leo et al., 2014).

Intriguingly, the TAAR1 agonist RO5166017 inhibited while the TAAR1 antagonist EPPTB promoted the firing rates of dopamine neurons in VTA and serotonin neurons in dorsal raphe nucleus (Revel et al., 2011). Moreover, dopamine neurons and serotonin neurons of TAAR1-KO mice showed increased firing rates (Revel et al., 2011). The firing activity of dopamine neurons from VTA also modulates dopamine release and transmission in the projecting areas, which could suggest an alternative mechanism that TAAR1 regulates dopamine transmission.

### TAAR1 modulates abused drug-induced dopamine transmission

Dopamine hypothesis in drug addiction was proposed over 40 years ago (Nutt et al., 2015). The mesocorticolimbic and striatonigral dopaminergic pathways are the most important dopaminergic pathways in the brain to regulate drug addiction. The fundamental role of TAAR1 within the mesocorticolimbic system in dopamine transmission seems to be a possible mechanism underlying the involvement of TAAR1 in drug addiction (Nutt et al., 2015).

Cotter et al. showed that the selective TAAR1 partial agonist RO5203648 inhibited METH-induced increase in extracellular dopamine level in the NAc *in vivo* (Cotter et al., 2015). However, RO5203648 did not affect METH-mediated inhibition of DA efflux and reuptake in striatal synaptosomes *in vitro*. Given that the *in vivo* data and *in vitro* results are not expected to be always consistent, these results might to some extent suggest that TAAR1 in the NAc but not dorsal striatum participated in METH-induced neurochemical alterations and behaviors. In another study, it was demonstrated that RO5203648 blocked cocaine-induced DA overflow in the NAc (Pei et al., 2014). Interestingly, RO5203648 did not change the clearance of DA, indicating that TAAR1 regulating cocaine-induced DA overflow by mechanisms other than interaction with DAT (Pei et al., 2014). Consistently, the TAAR1 full agonist RO5166017 did not affect DA clearance in the NAc (Leo et al., 2014). Behavioral studies also confirmed that RO5166017 decreased the hyperlocomotion in DAT-KO mice (Revel et al., 2011). Collectively, these results indicated that TAAR1 negatively modulates drug-induced dopamine accumulation in the NAc, which may be independent of DAT. As mentioned earlier, existing studies concerning the cellular mechanisms underlying TAAR1's action was primarily focused on DAT and D2-autoreceptors. Since DAT is probably not involved in TAAR1's action, D2-autoreceptors may be the molecule that mediates the inhibitory effects of TAAR1 on drug addiction-related behaviors.

## Current challenges in the understanding of TAAR1 and drug addiction

### Neural mechanisms that we don't know

Dopamine D2 receptors have two isoforms, short (D2_S_) and long (D2_L_) (Ford, 2014). The sequence differences between the two isoforms are within the third intracellular loop, which determines their distinct abilities to couple to G-proteins (Montmayeur et al., 1993). Recently, it was shown that TAAR1 could form heterodimers with D2 receptors but not with a GABA_B2_ receptor *in vitro* and in dopaminergic neurons from the VTA of mice overexpressing TAAR1 (Harmeier et al., 2015). The interaction between TAAR1 and D2 receptors increased the expression of TAAR1 on plasmid membrane (Harmeier et al., 2015). However, it should be noted that because of the low expression level of TAAR1 *in vivo*, the heterodimerization can only be detected in TAAR1-OE mice. Importantly, TAAR1-D2 receptor complex reduced TAAR1 agonist *p*-tyramine- and RO5166017-induced cAMP accumulation, which was blocked by a D2 receptor antagonist (Harmeier et al., 2015). These results indicated that the interaction between TAAR1 and dopamine D2 receptors dampened TAAR1 activation-induced cAMP-dependent pathway activity. These results raise an interesting question that needs to be addressed in the future: which isoform of D2 receptors can form heteromers with TAAR1? It is often suggested that the D2s isoform is the autoreceptors, but both D2s and D2L receptors may act as autoreceptors in dopaminergic neurons (Neve et al., 2013). Identifying the nature of the TAAR1-D2 receptor complex will help us to understand the mechanism underlying TAAR1's function.

**Figure 2:**
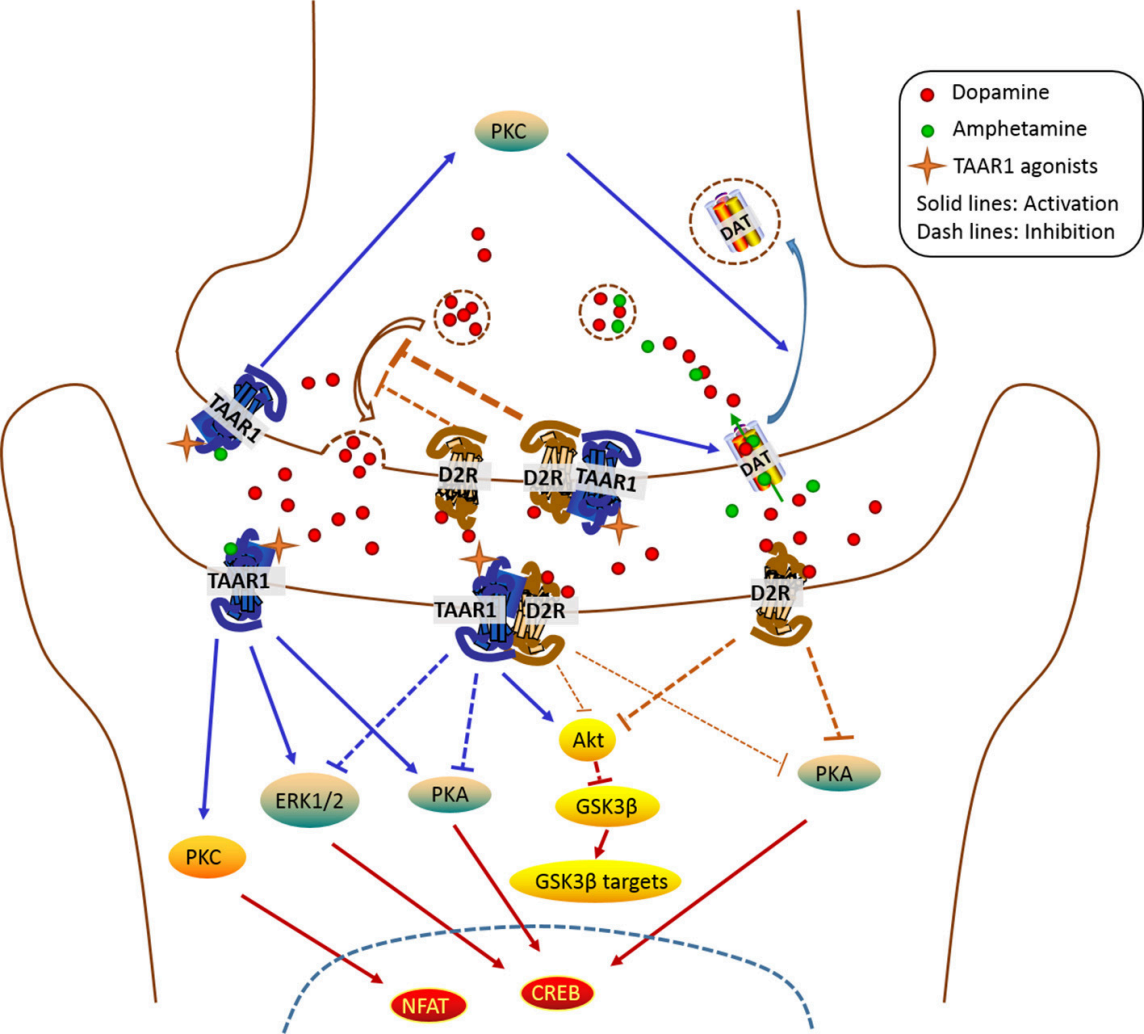
Schematic representation of the TAAR1 signaling pathways in the NAc. It should be noted that the subcellular distribution (presynaptic vs. post-synaptic; intracellular vs. membrane) of TAAR1 in the nucleus accumbens (NAc) is unclear. Modulation of DAT internalization by TAAR1 in the presynaptic terminal is dependent on PKC activation. TAAR1 enhances the function of presynaptic D2R while inhibiting post-synaptic D2R. In the post-synaptic membrane, TAAR1 activation-induced ERK1/2 and PKA/CREB signaling pathways are inhibited by the activation of TAAR1-D2R heterodimers. TAAR1 also signals through AKT/GSK3β pathway. TAAR1, trace amine-associated receptor 1; D2R, dopamine D2 receptors; PKA, protein kinase A; PKC, protein kinase C; ERK1/2, extracellular signal-regulated protein kinases 1 and 2; AKT, protein kinase B; GSK3β, glycogen synthase kinase 3β; CREB, cAMP response element binding protein; NFAT, nuclear factor of activated T-cells.

The *in vivo* setting is much more complex than the *in vitro* setting. The TAAR1-D2 complex could transduce through different signaling pathways *in vivo* from what we have known from *in vitro* studies. TAAR1 signaling cascade has been shown to involve PKA- and PKC-dependent pathways *in vitro* (Panas et al., 2012), as also demonstrated by the finding that TAAR1's effects on DAT were blocked by PKA and PKC antagonists (Xie and Miller, 2007). Theoretically, the consequence of TAAR1 activation would increase the phosphorylation levels of PKA- and PKC-dependent downstream signaling. However, when TAAR1 was coexpressed with D2 receptors, the TAAR1 activation-induced increase in phosphorylation of CREB and ERK was blocked. In contrast, interaction of TAAR1-D2 receptors enhanced TAAR1-induced β-arr2-dependent cascade, as revealed by the increase in phosphorylation levels of Akt and GSK3β (Harmeier et al., 2015). Furthermore, the phosphorylation levels of AKT and GSK3β decreased in the striatum of TAAR1-KO mice, suggesting an activation of D2 receptors/ AKT/GSK3β signaling pathway in TAAR1-KO mice (Espinoza et al., 2015). Both presynaptic and post-synaptic D2 receptors could be involved in a TAAR1's action *in vivo*. It was demonstrated that TAAR1 agonist potentiated quinpirole-induced inhibitory effect on DA release, suggesting that TAAR1 enhances presynaptic D2 receptors' function (Leo et al., 2014). However, activation of post-synaptic D2 receptors induced by quinpirole was increased in TAAR1-KO mice, suggesting that TAAR1 reduces post-synaptic D2 receptors' function (Espinoza et al., 2015). Collectively, it seems that, when forming heterodimers with D2 receptors, TAAR1 positively modulates presynaptic D2 autoreceptors while negatively regulating post-synaptic D2 receptors (Figure 2), however, such a relationship needs further characterization.

Dopaminergic projection in the NAc is originated from the VTA (Nutt et al., 2015). Systemic administration of TAAR1 agonists may also act on TAAR1 in the VTA to modulate dopamine transmission in the NAc (Liu et al., 2017). Interactions between TAAR1 and D2 receptors may explain the role of TAAR1 in the NAc in regulating dopamine accumulation of drugs, but whether it is a common mechanism that also occurs in the VTA needs further elucidation. Partial agonists and full agonists of TAAR1 regulate the firing rate of VTA neurons oppositely in *ex vivo* studies using brain slices. However, when assessed by pharmacologic magnetic resonance imaging (phMRI), a technique based on continuous arterial spin labeling, both the TAAR1 full agonist RO5256390 and the partial agonist RO5263397 decreased the blood perfusion of VTA in anesthetized rats (Revel et al., 2013b). It is possible that the activation of D2 autoreceptors in the soma and dendrites of dopaminergic neurons in the VTA induce dopamine efflux outside the synaptic cleft, which causes reduced dopamine release from presynaptic membrane.

Besides D2 receptors, other receptors may also be important players in the mediation of TAAR1 action. For example, D3 receptors have also been shown to interact with TAAR1. In a recent report, Siemian et al investigated the effects of TAAR1 agonists on quinpirole-induced yawning behavior (Siemian et al., 2017). Quinpirole produced an inverted U-shaped dose-effect curve in inducing the yawning behavior. The TAAR1 partial and full agonists RO5263397 and RO51669017 both inhibited the induction of quinpirole-induced yawning. Because it is well-characterized that a low dose of quinpirole induces yawning through D2 receptor activation while a high dose of quinpirole activates D3 receptor to counter the induction of yawning (Siemian et al., 2017), this apparent suppression of quinpirole-induced yawning behavior is most likely due to the indirect modulatory effects of TAAR 1 on D3 receptors. Many studies have shown that D3 receptors also play a critical role in regulating dopamine-related behaviors and drug addiction. It is crucial to clarify whether TAAR1 could interact with D3 receptors to modulate drug addiction.

### Clinical trials of TAAR1 agonists

Several clinical trials for TAAR1 agonists have been launched for the treatment of mental disorders. For example, a phase I clinical trial with a TAAR1 partial agonist RG7351 was initiated by Roche with the purpose to treat major depression (www.roche.com). Sunovion Pharmaceuticals Inc. also initiated a clinical study for SEP-363856, a dual agonist at 5-HT1A receptors and TAAR1, to treat schizophrenia. SEP-363856 is currently under phase II clinical trial to evaluate its safety, efficacy, and tolerability to treat schizophrenia (http://www.sunovion.us). If these clinical studies succeed, there would be exciting real possibility to test their efficacy for the treatment of drug addiction. This will be a long-awaited important development in the field of TAAR1 research.

### TAAR1 antagonist

The only selective TAAR1 antagonist currently available is EPPTB (RO5212773) (Bradaia et al., 2009). Pharmacological characterization suggests that EPPTB is a TAAR1 antagonist/inverse agonist (Stalder et al., 2011). EPPTB could increase the firing rate of DA neurons in the brain slice of VTA from WT mice but not from TAAR1-KO mice. EPPTB also prevented TAAR1 agonist *p*-tyramine (p-try)-induced reduction of the firing rate of DA neurons (Bradaia et al., 2009). The information on the *in vivo* pharmacological activities of EPPTB is sparse. Therefore, more systematic studies of EPPTB *in vivo* and the development of new TAAR1 antagonists would greatly boost the TAAR1 research. As an invaluable research tool, selective TAAR1 antagonists can be very useful in verifying the pharmacological selectivity of TAAR1 agonists (currently this can only be confirmed by transgenic cells or animals). In addition, selective antagonists would facilitate the understanding of endogenous TAAR1 ligands in regulating drug addiction. TAAR1 antagonists may also have therapeutic potential for certain neuropsychological disorders. TAAR1-KO mice demonstrated exaggerated degeneration of dopaminergic neurons and reduced response to I-DOPA, a compound restoring dopamine transmission and relieving symptoms of PD, suggesting that TAAR1 could be involved in the pathogenesis of PD (Alvarsson et al., 2015). In this regard, TAAR1 antagonists could be useful pharmacotherapies for dopamine deficiency disease, such as PD.

## Conclusion

TAAR1 negatively modulates drug addiction-related behaviors. The broad-spectrum inhibition of addiction-related effects of drugs of abuse by TAAR1 agonists suggests that TAAR1 agonists could potentially be valuable pharmacotherapy to treat drug addiction and relapse. Limited data also suggest that TAAR1 agonists could be useful to treat non-drug related behavioral addiction. The underlying mechanisms that TAAR1 modulates addiction-related behaviors have been fairly well established as primarily functionally regulating dopamine transmission. Growing evidence implicates that TAAR1 primarily acts through the interaction with both presynaptic and post-synaptic D2 receptors to synergistically control the downstream signaling pathways and the overall behavioral output. However, these are just the starting pieces of the puzzle and further studies are required to understand the detailed mechanisms of TAAR1 *in vivo*, which in turn may also drive the development of novel TAAR1-based pharmacotherapy to treat drug addiction.

## Author contributions

J-FL and J-XL planned and prepared the manuscript. Both authors agreed on the finalized version of the manuscript.

### Conflict of interest statement

The authors declare that the research was conducted in the absence of any commercial or financial relationships that could be construed as a potential conflict of interest.
